# Prognosis for ocular toxocariasis according to granuloma location

**DOI:** 10.1371/journal.pone.0202904

**Published:** 2018-08-30

**Authors:** Jin-woo Kwon, Sun Young Lee, Donghyun Jee, Yang kyung Cho

**Affiliations:** Department of Ophthalmology and Visual Science, St. Vincent’s Hospital, College of Medicine, Catholic University of Korea, Suwon, Republic of Korea; National Yang-Ming University Hospital, TAIWAN

## Abstract

**Purpose:**

To determine the prognosis for ocular toxocariasis (OT) according to the location of the granuloma and to identify factors associated with its recurrence within 1 year.

**Methods:**

OT patients were classified according to the granuloma lesion. After grouping the patients as posterior or peripheral, we compared sex, age, intraocular pressure, best corrected visual acuity (BCVA), degree of inflammation, immunoglobulin E, eosinophil profiles, recurrence, and complications in each group. We also identified factors associated with recurrence within 1 year.

**Results:**

A total of 29 (61.70%) patients had granuloma at the periphery, and 18 (38.30%) patients had granuloma around the posterior pole. There were no significant differences in ocular or systemic evaluations except the initial BCVA. The mean decimal BCVA of the posterior pole granuloma group was worse than that of the peripheral granuloma group (p = 0.042). After treatment, the mean BCVA of the posterior pole granuloma group improved significantly (p = 0.019), and the final mean BCVA was not significantly different between the groups (p = 0.673). Multiple logistic regression analyses revealed that recurrence within a year was associated with age at diagnosis (p = 0.007).

**Conclusions:**

The initial BCVA of OT patients differed according to the location of the granuloma, but the BCVA after treatment was not significantly different between the groups. Younger age was associated with recurrence within 1 year.

## Introduction

Toxocariasis is a parasitic disease involving infection by *Toxocara canis* or *Toxocara cati*.[1–3] The *Toxocara* species are clinically classified into visceral larva migrans when they involve the liver or lung, ocular larva migrans when they involve ocular inflammation, and neurological larva migrans when they involve the brain.[4,5]

Previous studies from Western countries have reported that ingestion of embryonated eggs or larvae through contact with infected puppies or contaminated soil was the main route of infection.[6–8] However, recent studies from Asia have reported a higher prevalence than expected in uveitis patients, whose infection may have involved ingestion of infected raw cow liver or meat.[9–14]

Ocular toxocariasis (OT) usually induces inflammation in uveal and retinal tissues, and results in diverse sequelae such as simple retinal pigmentation, epiretinal membrane (ERM), retinal nerve fiber layer (RNFL) defect, or macular scar.[9,10,13,15]

For the diagnosis of OT, clinical presentation and blood *Toxocara* IgG test results are widely used. The *Toxocara* IgG test is a method for measuring antibody titers using an indirect enzyme-linked immunosorbent assay (ELISA) based on *Toxocara* larvae crude antigen.[16,17] Although some studies revealed that ELISA test could have cross reactivity with other helminthes,[18,19] another study reported that the sensitivity and specificity were 91.5% and 91.0%, respectively.[20] Another study proved the usefulness of additional use of aqueous humor in ELISA test to improve accuracy of diagnosis.[21]

OT can manifest as peripheral or posterior pole granuloma and chronic panuveitis with no granuloma lesions.[22] In this study, we classified patients with OT granuloma into posterior pole and peripheral granuloma groups and compared them with regard to age, sex, serology, ocular manifestations, prognosis, and recurrence.

## Methods

The medical records of all naïve uveitis patients diagnosed with OT at St.Vincent’s Hospital, Suwon, Republic of Korea, between 2014 and 2017, were retrospectively reviewed. This study was performed according to the tenets of the Declaration of Helsinki, and the study protocol was approved by the Institutional Review Board and Ethics Board of the Catholic University of Korea and our hospital. Informed consent was not obtained because this study involved the review of patient records that were fully anonymized prior to the analyses.

All patients underwent a full ophthalmic examination, including measurements of best-corrected visual acuity (BCVA) expressed as decimal measured with Snellen chart, and intraocular pressure (IOP); a dilated fundus examination after maximum pupil dilation; a complete blood count; blood chemistry tests for *Toxocara* immunoglobulin (Ig)G, *Toxoplasma* IgM, and *Toxoplasma* IgG; an assessment of total IgE levels; chest X-rays; measurements of HLA-B27, angiotensin converting enzyme, antinuclear antibody, hepatitis B surface antigen antibody, and anti-hepatitis C virus antibody levels; a syphilis reagin test; and a questionnaire about eating habits and whether the patients had pets.

We included patients who had retinal granuloma and were seropositive for *Toxocara* IgG. After grouping patients by posterior pole or peripheral granuloma, we compared the groups on degree of vitreal and anterior segment inflammation, immunoglobulin IgE levels, eosinophil counts, eosinophilia (eosinophil counts >500/μL or >5.0% of the total white blood cell count), complications, recurrence within 1 year, IOP, and BCVA before and after treatment. We defined posterior pole granuloma group as granuloma lesions located inside major arcade or involved within the disc. Peripheral granuloma group was defined as granuloma located outside major arcade without disc involvement. The degree of vitiritis was classified using national institutes of health (NIH) grading system and that of anterior segment inflammation was classified using standardization of uveitis nomenclature working group grading scheme.[23,24]

All patients diagnosed with OT were treated with 30mg of oral prednisolone with tapering over 2 months, and with 400 mg albendazole twice per day for 2 weeks to minimize recurrence.[10]

Optical coherence tomography (OCT; Cirrus High Definition-OCT; Carl Zeiss Meditec, Dublin, CA, USA) was used to check for complications such as cystoid macular edema (CME) or ERM during the 1-year follow-up in OT patients.

The Wilcoxon signed-rank test was used to compare changes in IOP and BCVA. The Mann–Whitney U-test was used to compare age, IgE levels, eosinophil counts, BCVA, grade of inflammation, and IOP between two groups. The Fisher’s extract test was used to compare sex distribution, recurrence rate, numbers with eosinophilia, and complications in the same groups. After dividing all the patients into two groups according to whether recurrence showed within 1 year or not, the logistic regression test was used to identify parameters associated with recurrence. All statistical analyses were performed using SPSS statistical software for Windows, version 21.0 (SPSS, Chicago, IL, USA). Statistical significance was set at p < 0.05.

## Results

**Fig 1:**
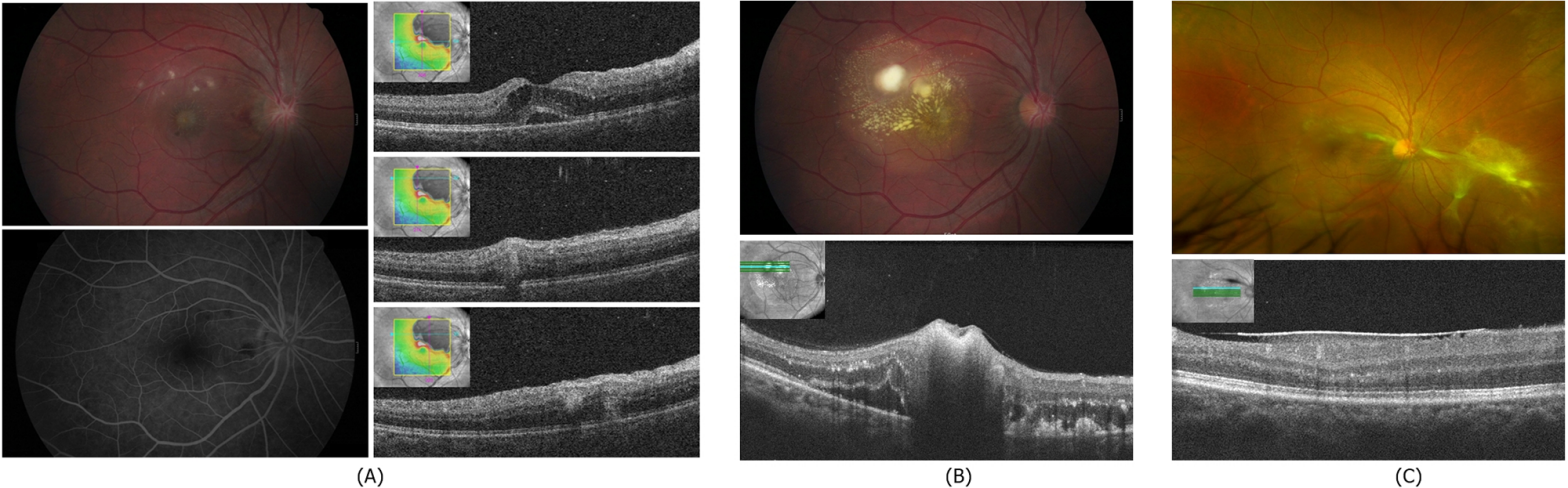
(A) Initial fundus photographs, FAG, and SD-OCT of a 34-year-old male who presented with suddenly decreased vision for 2 days in the right eye. The fundus finding in the right eye shows retinal hemorrhage around the disc edema, multiple retinal granulomatous lesions, and macular edema. Using FAG, there was no abnormal finding except blocked fluorescence at the granuloma and a hemorrhage lesion. SD-OCT shows that granulomas were located across the inner and outer retina. (B) Fundus photograph and SD-OCT at the 3-month recurrence. When compared with the initial visit, larger granulomas accompanying exudation around the lesion were present. (C) Fundus photograph and SD-OCT at 6 months after the initial visit. The location of granulomas changed to the nasal retina, but the ERM remained superior to the fovea. FAG, fluorescein angiography; SD-OCT, spectral domain-optical coherence tomography; ERM, epiretinal membrane.

Of 47 OT retinal granuloma patients, 29 (61.70%) had granuloma at the peripheral retina and 18 (38.30%) had granuloma at the posterior pole. The study group was composed of 38 males and nine females, and the average age was 53.06 ± 10.24 years. A total of 27 patients (57.45%) had a history of ingesting raw bovine liver. Five patients (10.64%) raised dogs or cats. Twelve patients (25.53%) had a recurrence of uveitis within 1 year of treatment. CME occurred in five patients, tractional retinal detachment (TRD) manifested in 14 patients, and ERM occurred in six patients. There were two cases of accompanying rhegmatogenous retinal detachment (RRD) with TRD, four cases of an optic disc involving granuloma, and one case of neuroretinitis at the initial manifestation (Fig 1).

**Fig 2:**
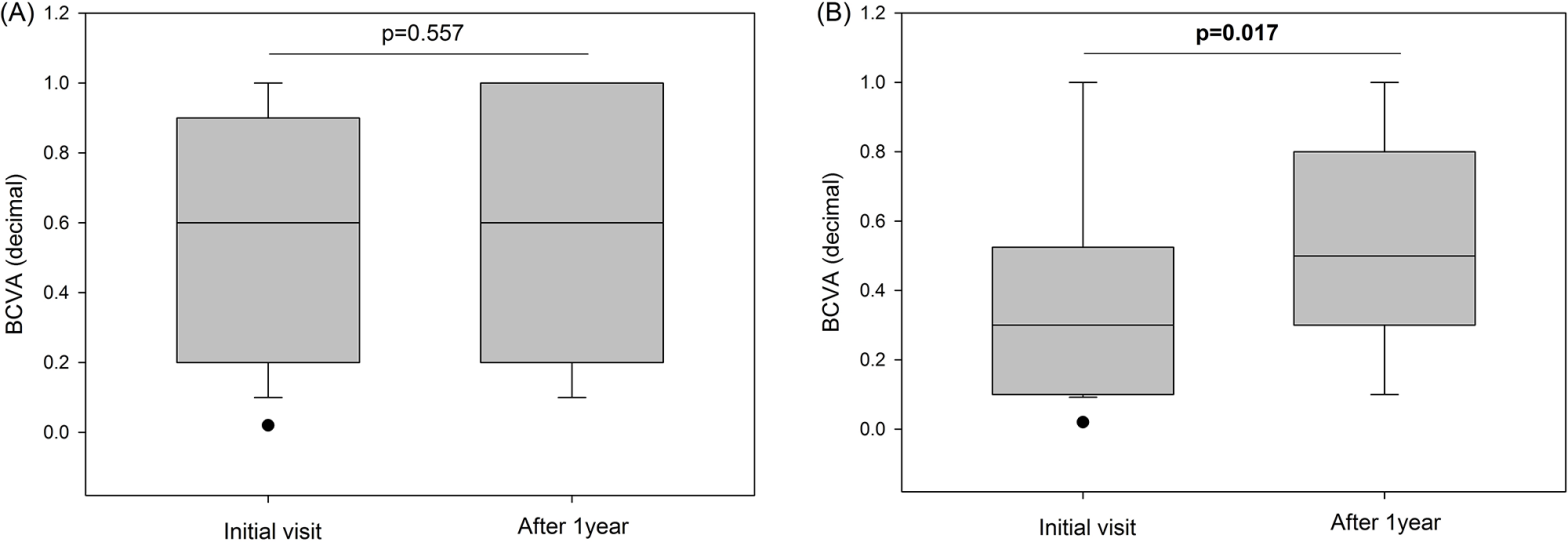
(A) Changes in best corrected visual acuity (BCVA) of the peripheral granuloma group. (B) Changes in BCVA of the posterior pole granuloma group. The BCVA increased after treatment with a significant difference in the posterior pole granuloma group (p = 0.017).

There were no significant differences between the two groups in ocular or systemic evaluations except for the initial BCVA. The mean decimal BCVA of the posterior pole granuloma group (0.362 ± 0.292) was worse than that of the peripheral granuloma group (0.583 ± 0.349; p = 0.042). After treatment, the BCVA of the posterior pole granuloma group improved significantly (0.539 ± 0.305; p = 0.019), and the final BCVA was not significantly different between the groups (p = 0.673; Table 1). The p values were slightly different in box plots when median values were compared instead of mean values (Fig 2).

**Table 1:** Comparison of the peripheral granuloma and posterior pole granuloma groups.

	Peripheral granuloma	Posterior pole granuloma	p value
**Demographics**			
n	29	18	
Male: female	22:7	16:2	0.449
Age (years)	52.38 ± 10.94	54.17 ± 9.17	0.677
**Initial ocular examination**			
BCVA (decimal)	0.583 ± 0.349	0.362 ± 0.292	0.042
IOP (mmHg)	15.52 ± 6.05	16.17 ± 8.03	0.692
Grade of vitreal inflammation (0~4+)	0.900 ± 0.618	1.083 ± 0.862	0.551
Grade of anterior inflammation (0~4+)	0.483 ± 0.688	0.333 ± 0.485	0.448
**1-year follow-up examination**			
BCVA (decimal)	0.586 ± 0.347	0.539 ± 0.305	0.673
IOP (mmHg)	13.45 ± 4.53	15.06 ± 3.30	0.059
Recurrence within 1 year	9 (31.03%)	3 (16.67%)	0.324
**Blood parameters**			
Total IgE (IU/mL)	390.97 ± 465.06	545.44 ± 701.77	0.896
Eosinophil counts (/μL)	182.83 ± 158.95	266.72 ± 236.30	0.437
Eosinophilia (>500/μL or >5.0% WBC)	5 (17.24%)	5 (33.33%)	0.473
**Complications**			
ERM	4 (13.79%)	2 (11.11%)	1.000
CME	3 (10.34%)	2 (11.11%)	1.000
TRD	11 (37.93%)	3 (16.67%)	0.191

BCVA, best corrected visual acuity; IOP, intraocular pressure; IgE, immunoglobulin E; WBC, white blood cells; ERM, epiretinal membrane; CME, cystoid macular edema; TRD, tractional retinal detachment.

### Parameters associated with recurrence according to logistic regression analyses

Univariate logistic regression analyses revealed that age and BCVA at diagnosis were associated with recurrence (OR 0.86, p = 0.002 and OR 15.64, p = 0.016, respectively). Multivariate logistic regression analyses showed that younger age was the only parameter associated with recurrence (OR 0.88, p = 0.007; Table 2).

**Table 2:** Variables associated with the recurrence of OT-related uveitis within 1 year according to logistic regression analyses.

	Univariate analyses*		Multivariate analyses*	
	Adjusted OR (95%CI)*	p value	Adjusted OR (95% CI)*	p value
Age	0.86 (0.77–0.94)	0.002	0.88 (0.79–0.95)	0.007
BCVA (initial visit, decimal)	15.64 (1.94–182.43)	0.016	6.70 (0.52–115.97)	0.154
IOP (initial visit, mmHg)	1.01 (0.91–1.11)	0.811		
Level of IgE (IU/mL)	1.00 (1.00–1.00)	0.621		
Eosinophil counts (/μL)	1.00 (1.00–1.01)	0.217		
Grade of vitreal inflammation (0–4+)^a^	1.60 (0.66–4.02)	0.281		
Grade of anterior inflammation (0–4+)^b^	1.13 (0.35–3.19)	0.823		

OR, odds ratio; CI, confidence interval; BCVA, best corrected visual acuity; IOP, intraocular pressure; IgE, immunoglobulin E.

*Adjusted for sex.

^a^ Classified using national institutes of health grading system

^b^ Classified using standardization of uveitis nomenclature working group grading scheme

## Discussion

A previous study reported that depending on the location of granuloma, different OT complications would occur: Granulomas at posterior pole were found to be associated with comorbidities like RNFL defects and ERM formations, while granulomas at periphery were with vitreous opacity.[10] However, that study did not report visual outcomes after treatment as a function of the location of the granuloma. We therefore analyzed prognoses for vision according to the location of the granuloma and then analyzed parameters associated with the recurrence of uveitis related to OT within 1 year.

We also compared parameters associated with the location of the granuloma to determine the activity or recurrence of OT. However, there were no significant differences in sex, age, initial IOP, IgE levels, eosinophil counts, BCVA or IOP at 1-year follow-up, degree of inflammation, or in recurrence and complications. The BCVA at the initial visit was the only parameter that differed significantly between the posterior pole and peripheral granuloma groups. The BCVA of the posterior pole group improved within 1 year, but the BCVA of the peripheral group was not significantly changed within 1 year, and the difference between the two groups was no longer significant.

The causes of no significant improvement of BCVA in peripheral OT granuloma group may be the lower severity of inflammation and remaining complications after treatments. The degree of vitreal inflammation was not severe according to the classification by NIH grade in this group. Additionally, there were only 11 patients with anterior uveitis out of 29 patients. We think that the effect of inflammation on BCVA was lower than expected. Even after treatment, cataract, ERM, and TRD with RRD also could make the mean VA change not to differ significantly.[25]

In the determination of IgE and eosinophil levels, only 59.57% of patients had IgE levels higher than the normal range (>100 IU/mL), and eosinophilia was present in only 21.28% of patients. These results suggest that IgE levels and eosinophilia are not appropriate screening markers for the diagnosis of OT.

The rate of recurrence was 31.03% in the peripheral granuloma group and 16.67% in the posterior pole granuloma group. The difference between the groups was not statistically significant. A previous study reported that recurrence at 6 months among patients with OT-related uveitis treated with antihelmintics and steroids was 17.4%.[10] The difference in these recurrence rates is mainly due to differences in the follow-up periods. Recurrence within 1 year was associated with younger age according to logistic regression analyses. Other parameters, including IOP, BCVA, IgE levels, degree of inflammation, and eosinophil counts, were not associated with recurrence within 1 year (Table 2), and the location of the granuloma did not differ between the groups (Table 1). One study about intermediate uveitis reported that patients who are 45 years or older had higher incidence of remission than those younger than 45 years (HR = 1.79).[26] The association between age and recurrence within 1 year in our study also suggests that OT infection at young age results in more recurrences and complications.

The OT patients were predominately male, and the OT patients in this Korean population were older than those in Western countries, where OT mainly causes pediatric uveitis due to contamination of the environment or infection from pets.[4,10,14,27–30] We suggest that these differences in sex and age distribution are the result of differences in the route of infection. Some adult males in Korea eat raw cow’s liver or meat, believing it to be good for ocular health, which may have been the cause of OT in previous studies.[10–12] Our study is consistent with these reports, showing a sex difference in the suspected route of infection. Of the 9 female OT patients, only 3 had a history of eating raw cow’s liver. However, of the 38 male OT patients, 24 had a history of eating raw cow’s liver.

This study has some limitations. The sample size was small, and the follow-up period was relatively short. In future studies, we plan to analyze additional cases over longer periods of up to several years after diagnosis. In addition, there may have been selection bias involving the residence of the study population, because our hospital is located in an urban area. A previous study reported that there was a difference in prevalence depending on location of residence,[10] but we did not examine this parameter. An accurate record of changes in residence locations may be helpful for tracing the infection route of the OT. In addition, other studies used aqueous humor as samples and western blot to confirm diagnosis of OT, which is more specific than ELISA with serum antibodies.[31] We detected IgG antibodies to *Toxocara* using only an indirect ELISA based on the *Toxocara* larva antigen from blood samples.[16,17] Analyses using additional samples or methods could have confirmed our results. Finally, although posterior pole granuloma group showed improvement on mean visual acuity, there might be visual field defect in cases with disc or foveal involvement. To compare accurate visual function, we should have performed visual field test.

In conclusion, we showed that the location of the granuloma was associated with initial visual acuity in OT patients but did not affect the prognosis for vision. We also showed that other ocular and systemic factors did not differ according to the location of the granuloma. Younger patients with OT exhibited a higher rate of recurrence in this study, so careful observation and frequent follow-ups are recommended for these patients.
