# Correction: Prognosis for ocular toxocariasis according to granuloma location

**DOI:** 10.1371/journal.pone.0207901

**Published:** 2018-11-15

**Authors:** Jin-woo Kwon, Sun Young Lee, Donghyun Jee, Yang kyung Cho

The following information is missing from the Funding section: This study was funded by the National Evidence-based Healthcare Collaborating Agency (Grant Number: HC16C2299). The funders had no role in study design, data collection and analysis, decision to publish, or preparation of the manuscript.
